# Association Between Inequality of Emergency Medical Supply Resources and In-Hospital Mortality in Patients With Acute Myocardial Infarction

**DOI:** 10.3389/ijph.2025.1608533

**Published:** 2026-02-05

**Authors:** Young Choi, Seoyoung Park, Kyoung Hee Cho

**Affiliations:** 1 College of Health Sciences, Catholic University of Pusan, Busan, Republic of Korea; 2 Department of Healthcare Service Management, College of Health and Medical Sciences, Sangji University, Wonju, Republic of Korea

**Keywords:** inequality, in-hospital mortality, acute myocardial infarction, disparities, emergency medical supply resources

## Abstract

**Objectives:**

This study aimed to investigate the relationship between regional inequality in emergency medical supply resources and in-hospital mortality among patients with acute myocardial infarction (AMI) in South Korea.

**Methods:**

We analyzed data from the Korean National Health Insurance Service claims database, focusing on 53,770 AMI patients admitted to emergency departments between 2012 and 2020. The inequality index of emergency medical supply resources was calculated based on the availability of emergency percutaneous coronary interventions (PCI) within each region.

**Results:**

Among 53,770 AMI patients, 4,840 (9.0%) died in-hospital. After adjusting for covariates, patients residing in areas with higher inequality indices had increased risk of in-hospital mortality compared to those in areas with the lowest inequality (index 0.50–0.75: HR 1.504, 95% CI 1.198–1.889; index ≥0.75: HR 1.689, 95% CI 1.493–1.910).

**Conclusion:**

This study highlights the importance of equitable distribution of emergency medical resources to reduce in-hospital mortality among AMI patients. Policymakers should prioritize strategies to address regional disparities in emergency medical supply resources to improve health outcomes.

## Introduction

Acute myocardial infarction (AMI), one of the conditions with a golden hour period [1], is a medical emergency characterized by a sudden blockage of blood flow to a section of the heart muscle, typically caused by a blood clot in the coronary artery [2]. The concept of the “golden hour” refers to the critical period immediately following a traumatic injury or the onset of a medical emergency, during which prompt medical treatment is most likely to prevent death or serious long-term effects [3, 4]. In recent decades, the mortality rate of AMI in South Korea has declined [5]. However, the in-hospital mortality rate for AMI in South Korea is reportedly approximately 5%–10% [6]; heart disease is the second leading cause of death in the country, with a mortality rate higher than that of other diseases [5].

Previous studies have emphasized that emergency resources are crucial for conditions in which treatment during the golden hour reduces mortality. Timely intervention [7] and skilled personnel [8] are the most important factors in treating these diseases, including AMI. Emergency medical services equipped with trained personnel and the necessary equipment can provide immediate care at the scene, stabilize patients, and improve survival rates [9]. In addition, rapid transportation to an appropriate medical facility should ensure that definitive care is initiated as soon as possible, thereby reducing the likelihood of complications [10–12]. Specialists who can provide timely treatment are crucial [13, 14]. Additionally, environmental factors, such as advanced medical equipment [9], effective emergency care systems [15], strategic resource allocation [16], and hospital preparedness to respond to emergency patients are important. From a policy perspective, it is important to note that regional inequality in emergency medical resources affects the mortality rate.

In most previous studies on the relationship between emergency medical supply resources and in-hospital mortality, regional emergency medical supply resources were defined as the number of emergency medicine doctors, specialists in specific fields, or emergency medical institutions in a region [17–21]. However, in cases of absence of emergency medical institution or specific specialists at the time of need and patient transfer to other emergency medical institutions, the number of emergency medical institutions or specialists in the region is only a nominal number and cannot be considered as an actual emergency medical resource.

The aim of this study was to define the inequality in regional emergency medical supply resources available as emergency medical resources when necessary and to identify the relationship between the inequality in regional emergency medical supply resources and in-hospital mortality in AMI.

## Methods

### Data Source

This study used data from the Korean National Health Insurance Service (KNHIS) claims database. Korea’s health insurance operates as a national health insurance system, and when any citizen uses medical services, medical institutions request that the KNHIS provide medical service usage details to the patient. This study used customized data provided by the KNHIS. Customized health information refers to the data that are processed and provided as customized data so that the health information data collected, held, and managed by the KNHIS can be used for policy and academic research purposes. This study included all patients who received emergency medical services between 2012 and 2020.

### Study Population

**FIGURE 1:**
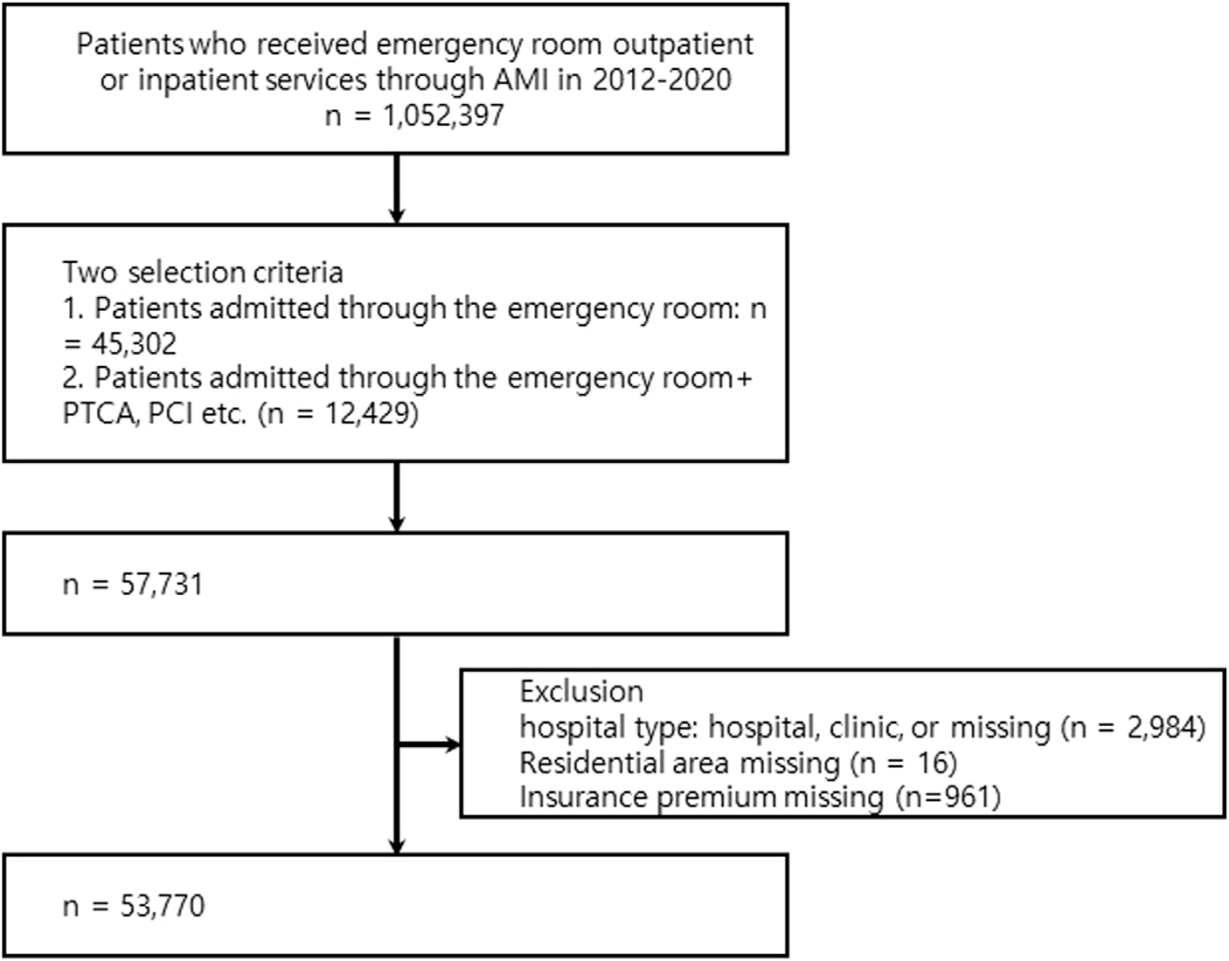
Flow chart of study population selection (Study on the association between inequality of emergency medical supply resources and in-hospital mortality, Republic of Korea, 2021–2024).

During 2012–2020, a total of 1,052,397 patients used emergency medical services for AMI. AMI was defined as a case billed with the 10th revision of the International Classification of Diseases (ICD) code I21.x as the main diagnostic code. This study targeted 57,731 of 1,052,397 patients with AMI admitted to the emergency department. Because the accuracy of the diagnosis codes in Korean claims data is approximately 70% [22], several exclusion criteria were used to define patients with true AMI. A total of 2,984 patients who used the emergency room for AMI and the type of medical institution was a hospital or clinic, 16 patients whose residence information was unavailable, and 961 patients whose health insurance premiums information was unavailable were excluded. Finally, 53,770 patients were included in this study (Figure 1). As this was a retrospective study using de-identified claims data provided by the KNHIS, the requirement for written informed consent was waived. The study protocol was reviewed and approved by the Institutional Review Board of Sangji University (1040782-210120-HR-01-74).

### Dependent Variable

In this study, in-hospital mortality was the dependent variable. We defined mortality to include all causes of death as identified from the death certificate data in the national death registry. For survival analysis, the observation period was defined as the duration of the AMI episode, starting from hospital admission with a primary diagnosis of AMI until discharge or in-hospital death. This definition ensures that the outcome measure captures mortality events directly associated with the patient’s hospitalization for AMI.

### Inequality Index of Emergency Medical Supply Resources by Region

The assessment of emergency medical supply resources was conducted using the number of emergency PCIs performed, with PCI performance identified as the key factor in reducing deaths in patients with acute myocardial infarction. These regions were divided into 256 administrative regions used in Korea, including cities, counties, and districts. To calculate the imbalance index of emergency medical supply resources by region, first, the number of PCIs performed as an emergency, not the number of planned PCIs, was calculated for each medical institution in each region. Then, the number of PCIs performed as an emergency was calculated by adding them up by region where the medical institution was located. In addition, the number of cases in which patients who visited a medical institution through the emergency room for AMI were transferred to a medical institution in another region and underwent PCI as an emergency was calculated. For example, assuming that a patient resides in Region A, the number of cases in which PCI was performed as an emergency by a medical institution located in Region A and the number of cases in which patients visited a medical institution for acute myocardial infarction but were transferred to a medical institution located in another region and performed as an emergency were calculated. The denominator was the number of cases in which PCI was performed as an emergency by a medical institution in each region plus the number of cases in which PCI was performed as an emergency by a medical institution located in another region; the numerator was the number of cases in which PCI was performed as an emergency by a medical institution located in another region. If a patient was transferred to another medical institution and underwent emergency PCI but the medical institution to which the patient was transferred was in the patient’s residential area, it was not included as a case performed in another region. The index value ranges from 0 to 1; the closer it is to 1, the more the number of cases in which PCI was not performed in the patient’s residential area, which may be because of lack of medical supply resources for performing PCI in the region. This index was calculated based on patient residence and was divided into four categories as absolute values.

$$\text{Inequality index of emergency medical supply resources by region}=\frac{\text{The}\;\text{number}\;\text{of}\;\text{cases}\;\text{in}\;\text{which}\;\text{PCI}\;\text{was}\;\text{performed}\;\text{as}\;\text{an}\;\text{emergency}\;\text{in}\;\text{another}\;\text{region}\;\text{than}\;{\text{patient}}^{\prime }s\;\text{residence}}{\text{The}\;\text{number}\;\text{of}\;\text{cases}\;\text{in}\;\text{which}\;\text{PCI}\;\text{was}\;\text{performed}\;\text{as}\;\text{an}\;\text{emergency}\;\text{in}\;{\text{patient}}^{\prime }s\;\text{residence}+\text{The}\;\text{number}\;\text{of}\;\text{cases}\;\text{in}\;\text{which}\;\text{PCI}\;\text{was}\;\text{performed}\;\text{as}\;\text{an}\;\text{emergency}\;\text{in}\;\text{another}\;\text{region}\;\text{than}\;{\text{patient}}^{\prime }s\;\text{residence}}$$

### Covariates

The covariates for our study were age (≤39, 40–49, 50–59, 60–69, or ≥70 years), sex, health insurance premium (medical aid, Q1, Q2, Q3, Q4), residential area (city, county, and borough), whether to transfer (yes or no), Charlson Comorbidity Index [23](CCI) (0, 1, 2, or ≥3), disability (yes or no), whether PCI is performed (yes, or no), and type of medical institution (general or tertiary). The Korean health insurance system is divided into medical aid and health insurance, and in the case of low-income earners below a certain level, they are classified as medical aid and are operated as taxes. In the case of health insurance, premiums are paid based on income. In our study, health insurance premium level was used as a proxy variable for patient income. CCI was used to reflect patient complexity.

### Statistical Analysis

Descriptive statistics were calculated for all the variables. The chi-squared test was used to evaluate statistically significant difference in the proportion of categorical variables. The survival probability for all-cause mortality was estimated using the Kaplan–Meier product limit method, and the log-rank test was used to stratify the inequality of the emergency medical supply resource index. To investigate the association between inequality emergency medical supply index and in-hospital mortality, we performed survival analyses using Cox proportional hazards regression. The proportional hazards assumption of the Cox regression models was formally tested using Schoenfeld residuals, and no evidence of violation was observed. All statistical analyses were performed using SAS 9.4 software.

## Result

**FIGURE 2:**
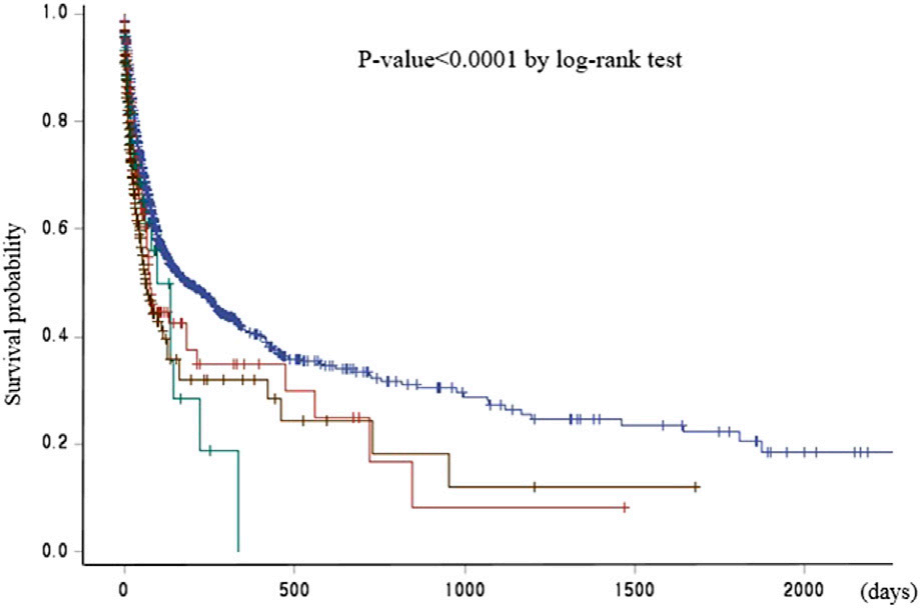
Survival probability by inequality in emergency medical supply resources (Study on the association between inequality of emergency medical supply resources and in-hospital mortality, Republic of Korea, 2021–2024).

Of 53,770 patients who visited the emergency room for AMI, 4,840 (9.0%) died and 48,930 (91.0%) survived (Table 1). Significant differences were observed between the two groups in all individual patient characteristics (age, sex, level of health insurance, residential area, CCI, whether transferred, disability, whether underwent PCI, and type of hospital). Kaplan–Meier analysis showed that the mean survival time decreased as the inequality in emergency medical supply resource index increased (p < 0.0001 by log-rank test; Figure 2). The information on residential area, whether transferred, CCI, disability, whether underwent PCI, and hospital type is shown in Table 1. Table 2 presents the in-hospital mortality results of the Cox proportional hazards regression after controlling for all covariates, including age, sex, health insurance premium level, residential area, whether transferred, CCI, disability, whether underwent PCI, and type of hospital. The closer the inequality index of emergency medical resources is to 1, the more likely it is that PCI was performed in the patient’s residential area and that patients will be transferred to another area. This index was divided into four categories based on absolute values, and the region corresponding to an inequality index of 0.25 or less, that is, the region with the fewest patient transfers, was used as the reference group. Compared to the reference group, the in-hospital mortality hazard ratio of patients residing in areas with inequality index values of 0.26–0.5 was 1.084 (95% CI, 0.952–1.235), with values of 0.5–0.75 was 1.504 (95% CI, 1.198–1.889), and t with values of 0.75 or higher was 1.689 (95% CI, 1.493–1.910), respectively.

**TABLE 1:** Characteristics of the study population, 2012–2020. (Study on the association between inequality of emergency medical supply resources and in-hospital mortality, Republic of Korea, 2021–2024).

	Total	Alive		Dead		p-value
Characteristics	N = 53,770	N = 48,930	(91.0)	N = 4,840	(9.0)	
Age, n (%)						
<39	1,519	1,493	(98.3)	26	(1.7)	<0.0001
40–49	4,654	4,542	(97.6)	112	(2.4)	
50–9	10,102	9,729	(96.3)	373	(3.7)	
60–69	11,748	11,041	(94.0)	707	(6.0)	
>70	25,747	22,125	(85.9)	3,622	(14.1)	
Sex, n (%)						
Male	35,757	33,032	(92.4)	2,725	(7.6)	<0.0001
Female	18,013	15,898	(88.3)	2,115	(11.7)	
Health Insurance Type, n (%)						
Medical aid	5,566	4,910	(88.2)	656	(11.8)	<0.0001
Health Insurance (Q1)	9,557	8,686	(90.9)	871	(9.1)	
Health Insurance (Q2)	8,881	8,175	(92.1)	706	(7.9)	
Health Insurance (Q3)	11,358	10,476	(92.2)	882	(7.8)	
Health Insurance (Q4)	18,408	16,683	(90.6)	1,725	(9.4)	
Patient’s residential area, n (%)						
Si (City)	24,109	21,937	(91.0)	2,172	(9.0)	0.0669
GUN (county)	7,525	6,898	(91.7)	627	(8.3)	
GU (borough)	22,136	20,095	(90.8)	2,041	(9.2)	
Transfer						
No	39,571	36,098	(91.2)	3,473	(8.8)	0.0024
Yes	14,199	12,832	(90.4)	1,367	(9.6)	
Charlson’s comorbidity index, n (%)						
≤1	1,479	1,394	(94.3)	85	(5.7)	<0.0001
2	25,474	23,414	(91.9)	2,060	(8.1)	
3	18,785	17,125	(91.2)	1,660	(8.8)	
>=4	8,032	6,997	(87.1)	1,035	(12.9)	
Disability, n (%)						
No	40,852	37,605	(92.1)	3,247	(7.9)	<0.0001
Yes	12,918	11,325	(87.7)	1,593	(12.3)	
Performing intervention, n (%)						
No	44,606	40,504	(90.8)	4,102	(9.2)	0.0005
Yes	9,164	8,426	(91.9)	738	(8.1)	
Hospital type, n (%)						
Tertiary hospital	29,058	26,906	(92.6)	2,152	(7.4)	<0.0001
General Hospital	24,712	22,024	(89.1)	2,688	(10.9)	
Inequality Index, n(%)						
<0.25	49,735	45,521	(91.5)	4,214	(8.5)	<0.0001
0.25–0.49	2,125	1,866	(87.8)	259	(12.2)	
0.50–0.74	484	407	(84.1)	77	(15.9)	
≥0.75	1,426	1,136	(79.7)	290	(20.3)	

**TABLE 2:** Hazard ratio for all-cause in-hospital mortality acute myocardial infarction patients admitted through the emergency room during 2012–2020 (Study on the association between inequality of emergency medical supply resources and in-hospital mortality, Republic of Korea, 2021–2024).

	Unadjusted		Adjusted	
Characteristics	HR	95% CI	HR	95% CI
Age				
<39	1.00		1.00	
40–49	1.20	(0.78–1.84)	1.19	(0.77–1.82)
50–9	1.68	(1.13–2.500	1.68	(1.13–2.51)
60–69	2.50	(1.69–3.70)	2.61	(1.77–3.87)
>70	4.86	(3.31–7.15)	5.27	(3.58–7.77)
Sex				
Male	1.00		1.00	
Female	1.27	(1.20–1.35)	0.95	(0.89–1.00)
Health Insurance Type				
Medical aid	1.07	(0.98–1.17)	1.09	(1.00–1.20)
Health Insurance (Q1)	0.98	(0.90–1.06)	1.18	(1.02–1.20)
Health Insurance (Q2)	0.90	(0.83–0.98)	1.09	(1.00–1.19)
Health Insurance (Q3)	0.87	(0.80–0.95)	0.97	(0.90–1.06)
Health Insurance (Q4)	1.00		1.00	
Patient’s residential area				
Si (City)	1.13	(1.03–1.23)	1.19	(1.19–1.42)
GUN (county)	1.00		1.00	
GU (borough)	1.12	(1.02–1.22)	0.49	(0.49–0.56)
Transfer				
No	1.00		1.00	
Yes	0.54	(0.51–0.58)	0.52	(0.49–0.56)
Charlson’s comorbidity index				
≤1	1.00		1.00	
2	1.25	(1.00–1.55)	1.17	(0.95–1.46)
3	1.18	(0.95–1.46)	1.03	(0.83–1.29)
>=4	1.16	(0.93–1.45)	1.05	(0.84–1.32)
Disability				
No	1.00		1.00	
Yes	1.23	(1.16–1.31)	1.23	(1.15–1.31)
Performing intervention				
No	1.00		1.00	
Yes	0.99	(0.92–1.08)	1.03	(1.04–1.22)
Hospital type				
Tertiary hospital	1.00		1.00	
General Hospital	1.42	(1.34–1.50)	1.29	(1.21–1.37)
Inequality Index				
<0.25	1.00		1.00	
0.25–0.49	1.41	(1.24–1.60)	1.08	(0.95–1.24)
0.50–0.74	1.89	(1.51–2.37)	1.50	(1.20–1.89)
≥0.75	2.14	(1.90–2.41)	1.69	(1.49–1.91)

## Discussion

In this study, we defined the imbalance in regional emergency medical supply resources available to patients with AMI who visited the emergency room as the extent to which PCI could be performed within the region without transfer to another region when needed. Furthermore, we investigated the relationship between the imbalance in emergency medical supply resources and in-hospital death. The aim of this study was to assess the actual imbalance in regional emergency medical supply resources and clarify the relationship between the actual imbalance and in-hospital death. We found that an increased necessity for patients to be transferred to another region due to the inability to perform PCI locally is associated with a higher risk of in-hospital death for patients living in that region. In addition to the degree of PCI performed in the region, age, sex, patient residence type, whether they were transferred, whether they underwent PCI, and hospital type were associated with the risk of in-hospital death.

AMI is a condition that can be managed effectively during the golden hour. The most important factor in reducing the risk of death for patients is performing PCI, which can open the blocked coronary artery within appropriate time [1, 10]. Most previous studies have simply defined emergency medical resources as the number of specialists, facility equipment personnel, etc., and have revealed their relationship with in-hospital deaths [9, 14, 17]. However, even if there are many specialists in a region who can perform PCI, if they cannot be utilized when PCI is needed, they are only nominal emergency medical resources and not actual emergency medical supply resources. In this study, it was found that the risk of in-hospital death increased in patients living in regions where PCI was not performed and where there was a high degree of transfer to other regions. There are two potential explanations for this finding. First, if the patients’ symptom duration and myocardial infarction severity were the same, PCI should have been performed within appropriate time; however, it is possible that they were transferred to other regions and did not receive appropriate intervention within an appropriate time. Second, if PCI was not performed in the region and the patients were transferred to other regions, there is a possibility that the region’s PCI performance experience and skill level of PCI performance would be low, which could increase the patient’s risk of death. In addition, in areas with insufficient emergency medical supply resources, there is also a possibility of a shortage of resources necessary for patients, such as a lack of resources necessary for transport in addition to emergency medical supply resources, or a lack of facilities, equipment, and personnel that can cover the severity of the patient’s condition.

In our study, the risk of in-hospital death was lower in transferred patients, which is inconsistent with the results of previous studies [24–27]. Because our analysis was based on claims data, we were unable to account for various factors such as the timing of the patients’ symptom onset, the time from the onset of symptoms to arrival at the medical institution, whether interventions were performed during that period, the number of blocked coronary arteries, the degree of blockage (complete or partial), and other clinical severities related to myocardial infarction. Therefore, we believe that these factors may have influenced our results. Furthermore, according to our findings, transfer reflects the severity of the patient’s AMI. If the severity of myocardial infarction was severe upon arrival at the medical institution, there is a possibility that the patient died without the opportunity to transfer. In other words, we believe that this result can be attributed to the less severe nature of AMI in the case of transferred patients, allowing them to be transferred, whereas more severe cases resulted in death. Interestingly, our study found that transferred patients had lower in-hospital mortality compared to those who were not transferred, which appears counterintuitive given that transfer is generally associated with delayed treatment and worse outcomes in AMI. While this finding may reflect selection bias, with more stable patients being transferred and more severe cases dying before transfer, this hypothesis cannot be directly tested with the available claims data. Moreover, due to the expiration of our data access period, we were unable to conduct further subgroup or interaction analyses to explore whether the association between transfer and mortality varies across levels of the inequality index. This limitation highlights the need for future studies with extended data access to investigate this issue in greater depth.

Our study has several limitations. First, our study used claims data, and the accuracy of the primary diagnosis code in the claims data in Korea is 70% [22]. Therefore, it was essential to establish a process to identify “true” AMI cases within the claims dataset. Among a total of 1,052,397 patients with an AMI diagnosis code, we restricted our analysis to those admitted via the emergency department (n = 45,302) and, among them, those who underwent interventional procedures such as PTCA or PCI (n = 12,429). We further excluded cases from clinics (n = 2,984) and patients with missing values in insurance type or residential information (n = 977). Through this process, we derived the final analytic cohort of 53,770 patients. This approach was intended to ensure that our analysis focused on patients with a high likelihood of being true AMI cases, rather than those with suspected AMI who might have been discharged after minimal treatment. However, we acknowledge that this analytic cohort may not fully represent the entire AMI population in South Korea, since some cases (e.g., patients who died upon arrival at the emergency department) may not have been adequately captured. In addition, because our access to the dataset has expired, we were unable to perform further comparative analyses between the analytic cohort and the full AMI population. These limitations should be considered when interpreting our findings, as they may affect the generalizability of the results to all AMI patients. Second, we were unable to consider the various factors that occurred during the prehospital period and their impact on in-hospital death. For example, we were unable to assess the timing of the onset of symptoms, the duration between symptom onset and arrival at the medical institution, or the treatment received during that period. In addition, because we used claims data, we could not determine disease severity in patients with AMI. We could not reflect upon clinically relevant factors, such as the type of AMI, number of occluded coronary arteries, degree of occlusion, and patient’s vital signs. Nevertheless, our study has several strengths. First, the analysis used sample data from the patients who visited the emergency room but rather the entire dataset of patients who visited the emergency room for AMI. Therefore, errors caused by sampling could be reduced. Second, the imbalance of regional medical supply resources was assessed based on the actual amount of resources available in an emergency, rather than relying on nominal resources, such as facilities, equipment, and personnel. This is expected to be particularly useful when measuring the supply of resources for conditions, such as AMI that require treatment within the golden hour. Although our study benefited from a large national dataset, the very large sample size may also increase the likelihood that even very small differences or weak associations reach statistical significance. Therefore, our findings should not be interpreted solely based on p-values. Instead, the magnitude of effect sizes and their clinical implications should be given greater weight. In this regard, we emphasize that some associations, while statistically significant, showed relatively small effect sizes, and thus their clinical impact may be limited. This highlights the importance of cautious interpretation when translating statistically significant results into clinical or policy recommendations. Third, although we included several individual-level covariates in the models, the possibility of residual confounding remains. In particular, unmeasured regional characteristics may influence both the inequality index and mortality outcomes, which could bias the observed associations. Moreover, because the exposure was defined at the regional level, the results may be subject to ecological bias and should therefore be interpreted with caution when inferring individual-level risks.

### Conclusion

Numerous studies have shown a correlation between medical supply resources and health outcomes, such as patient death or readmission. Most of these previous studies assessed supply resources by quantifying the number of beds per 100 patients, doctors, or nurses. However, for conditions that require treatment within the golden hour, the assessment of available medical resources in emergency situations should include the availability of essential resources for treating the illness, such as specialized medical staff or other necessary hospital resources. This will provide a more precise evaluation of medical resources in emergency situations. This assessment will be useful for estimating the emergency medical supply resources needed by a region or establishing policies for distribution. In addition, to reduce in-hospital deaths of patients with AMI, it is necessary to investigate the factors leading to patient transfer to other regions for PCI and to formulate strategies for the training and deployment of PCI specialists.
